# Electromyographic activation patterns during swallowing in older adults

**DOI:** 10.1038/s41598-021-84972-6

**Published:** 2021-03-11

**Authors:** Jin Young Ko, Hayoung Kim, Joonyoung Jang, Jun Chang Lee, Ju Seok Ryu

**Affiliations:** Department of Rehabilitation Medicine, Seoul National University Bundang Hospital, Seoul, National University College of Medicine, 82 Gumi-ro 173 Beon-gil, Bundang-gu, Seongnam-si, Gyeonggi-do 463-707 South Korea

**Keywords:** Ageing, Medical research, Geriatrics

## Abstract

Age-related weakness due to atrophy and fatty infiltration in oropharyngeal muscles may be related to dysphagia in older adults. However, little is known about changes in the oropharyngeal muscle activation pattern in older adults. This was a prospective and experimental study. Forty healthy participants (20 older [> 60 years] and 20 young [< 60 years] adults) were enrolled. Six channel surface electrodes were placed over the bilateral suprahyoid (SH), bilateral retrohyoid (RH), thyrohyoid (TH), and sternothyroid (StH) muscles. Electromyography signals were then recorded twice for each patient during swallowing of 2 cc of water, 5 cc of water, and 5 cc of a highly viscous fluid. Latency, duration, and peak amplitude were measured. The activation patterns were the same, in the order of SH, TH, and StH, in both groups. The muscle activation patterns were classified as type I and II; the type I pattern was characterized by a monophasic shape, and the type II comprised a pre-reflex phase and a main phase. The oropharyngeal muscles and SH muscles were found to develop a pre-reflex phase specifically with increasing volume and viscosity of the swallowed fluid. Type I showed a different response to the highly viscous fluid in the older group compared to that in the younger group. However, type II showed concordant changes in the groups. Therefore, healthy older people were found to compensate for swallowing with a pre-reflex phase of muscle activation in response to increased liquid volume and viscosity, to adjust for age-related muscle weakness.

## Introduction

The incidence of dysphagia has been known to increase with age^1^. Dysphagia increases the risk of aspiration and is known to induce pneumonia, which increases morbidity and mortality among older adults^2,3^. Anatomically, sarcopenia occurs due to the aging process, which causes atrophy and fatty changes in the striated muscles, including the lingual muscles^4,5^. In addition, sensory-motor function is reported to be decreased due to changes in peripheral and central nerve conduction in old age^6^. This neurological change was reported to slow the swallowing movement and increase cortical activation due to effortful swallowing^7,8^. Functionally, these neuroanatomical changes lead to a decrease in pharyngeal and tongue strength, which is directly related to the aspiration status in healthy older adults^9,10^.

Recently, coordinated movement of the oropharyngeal muscles and the opening mechanism of the upper esophageal sphincter (UES) have been actively studied in the kinematic analysis of a videofluoroscopic swallowing study (VFSS) and high-resolution manometry (HRM)^11–13^. Several studies have also been conducted to reveal the electrophysiological changes in the swallowing process in older adults using surface electromyography (S-EMG). These studies showed that the activation duration of the oropharyngeal muscles increases with aging^12,14,15^; however, despite this increase in the duration of activation of the muscles, kinematic analysis did not show an increase in duration^16^. Thus, it can be inferred that muscle activation is a protective mechanism so that neuromuscular degeneration does not lead to kinematic and functional loss. However, little is known about these protective mechanisms in the individual swallowing-related muscles of older adults. If we can systematically analyze and understand the activation pattern of each swallowing-related muscle, we will be able to identify changes in the swallowing process due to aging. Furthermore, this could lead to the development of a method to prevent and treat age-related dysphagia.

Firstly, we hypothesized that there is a protective mechanism that helps to adjust the anatomical and functional loss in older adults. This is thought to be the result of enhanced activity of swallowing-related muscles, which is a mechanism for neuromuscular adaptation. We assumed that this increase in muscle activity would be observed with S-EMG findings such as an increase in the amplitude and a prolongation in duration. Secondly, we also hypothesized that age-related changes in muscle activation patterns will differ depending on the volume and viscosity of the swallowed liquid. We assumed that the duration and amplitude of muscle activation are increased further when a large volume of liquid and/or a highly viscous liquid is swallowed.

Therefore, the purpose of this study was to find out the age-related differences in muscle activation patterns regarding the bolus volume and viscosity, and the possible protective mechanisms required to adjust the aging process.

## Materials and methods

### Participants

This study was a prospective, experimental study conducted from July 4, 2016, to November 21, 2017. It was performed at the rehabilitation unit of tertiary hospitals.

The inclusion criteria were age greater than 19 years, having no underlying diseases, having stable vital signs, willing to participate in the present study, and provision of informed consent. To screen for symptoms of dysphagia, each participant completed a 3-oz water swallow test^17^. During two trials of cup drinking, a trained research assistant observed the participants to determine if the following symptoms were present: choking, coughing, throat clearing, or wet voice. Patients with the following were excluded: (a) symptoms of dysphagia such as difficulty chewing, coughing, and hoarseness related with swallowing, food residues, and past history of pneumonia, (b) history of previous neck surgery, (c) presence of a neuromuscular disorder, (d) presence of a peripheral neuropathy and cranial neuropathy, and (e) history of brain disorders such as stroke, brain tumor, and cerebral palsy. Adults aged over 60 years were classified into the older group.

The study protocol was approved by the Seoul National University Bundang Hospital institutional review board (IRB No.: B-1604/344-003) and was registered at clinicaltrial.gov (Registration number: NCT03494361, Initial release: 2/04/2018, Last release: 10/04/2018, Actual study start date: 7/04/2016, Actual study completion date: 11/21/2017). Furthermore, all methods were performed in accordance with the relevant guidelines and regulations.

### Experimental procedures

All subjects were seated on a comfortable chair and instructed to complete the swallowing tasks. Firstly, S-EMG was recorded in a resting state, which was used as the baseline for normalization. During the test, the participants were required to keep their heads in a neutral position and avoid any movement. Then, the participants were instructed to swallow three types of boluses, twice for each type, in the following order: water 2cc, water 5cc (International dysphagia diet standardization initiative (IDDSI; level 0), and yogurt 5cc (IDDSI, level 4)^18^. Each bolus was given in an identical syringe. During the measurements, the participants held the liquid in their mouths and then swallowed when the examiner instructed them to do so. There was a 3-minute interval between each swallowing task. All liquids were administered by one therapist. S-EMG was recorded during the swallowing of each liquid. All measured data were analyzed, except data with severe artifacts.

### S-EMG signal acquisition

**Figure 1:**
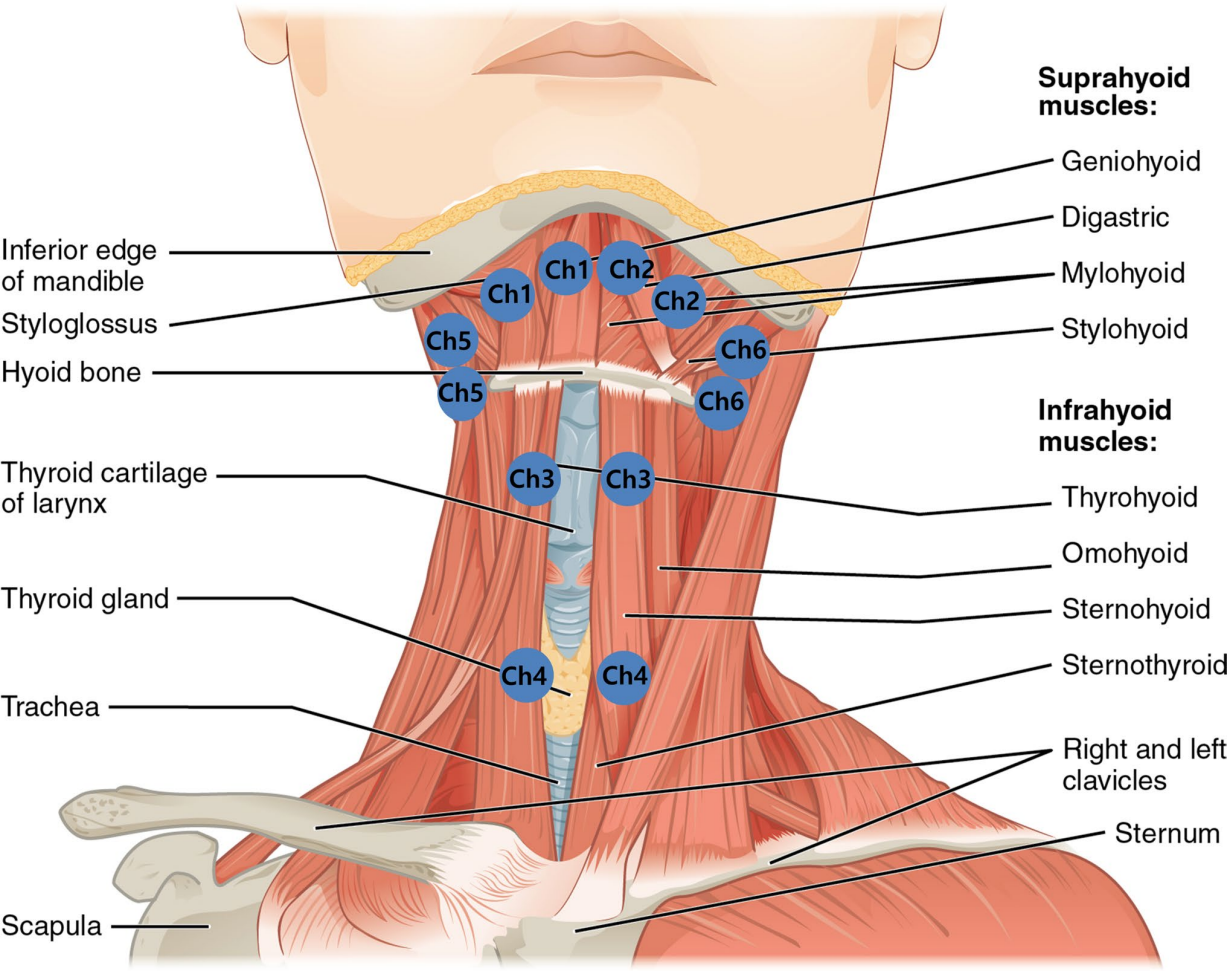
Locations of S-EMG electrodes (channel 1, 2: bilateral right suprahyoid muscles, channel 3: thyrohyoid muscle, channel 4: sternohyoid muscle, channel 5, 6: bilateral retrohyoid muscles). The copyright owner is OpenStax (Source: https://cnx.org/contents/FPtK1zmh@8.25:fEI3C8Ot@10/Preface). JS Ryu recreated the drawing by adding electrodes to the original drawing).

To maintain consistency, one experienced therapist (HY Kim) conducted the tests. In order to reduce the skin resistance, we wiped the skin with alcohol before attaching the electrodes. The six-channel surface electrodes were placed over the bilateral SH, bilateral retrohyoid (RH), thyrohyoid (TH), and sternothyroid (StH) muscles (Fig. 1). Electrode positions were determined using anatomical landmarks and manual palpation. To confirm proper placement of the electrodes, ultrasound examination was used to minimize muscle overlap, which can cause the crosstalk effect during S-EMG. Channel 1 (right) and channel 2 (left) electrodes were placed superior to the hyoid bone and posterior to the mandible with a 1-cm interval from the midline to target the digastric (anterior belly) and mylohyoid muscles. Channel 3 electrodes were placed on the bilateral superior pole of the thyroid cartilage to target the TH muscles, and channel 4 electrodes were placed medial to the sternocleidomastoid muscle and inferior to the thyroid cartilage to target the sternohyoid, omohyoid, and StH muscles. Channel 5 (right) and 6 (left) electrodes were attached just behind the mandible and in front of the sternocleidomastoid muscle to target the posterior belly of the diagastric and stylohyoid muscles (RH muscles)^19^.

To evaluate the degree of muscle contraction during swallowing, a wireless S-EMG analysis system (BTS FREEEMG 1000 with EMG-BTS EMG-Analyzer, BTS Bioengineering Co.) was used for the electrophysiological quantitative analysis. S-EMG signals were received from the six channels simultaneously, converted into a digital signal, and transmitted to a personal computer wirelessly. The acquisition frequency of the S-EMG signal was 1024 Hz and we used a band pass filter between 20 and 500 Hz. We quantitatively assessed the onset latency, duration, and peak amplitude of the contraction of the SH, RH, TH, and StH muscles.

### S-EMG analysis of the activation patterns and sequence

Because we predicted an increase in muscle activation, we analyzed the duration and amplitude of activation in each swallowing muscle. While analyzing the S-EMG data, we could identify unpredicted and important points in every recorded muscle; the activation pattern was largely divided into two types in both the young and older groups. The entire S-EMG signal was re-analyzed by a physician and another researcher. They agreed on how to divide the S-EMG graph pattern before analyzing the S-EMG signal. Since the S-EMG signal analyzer automatically analyzed the raw data values of the S-EMG signal as a two-dimensional curve, it was not difficult to divide the curve pattern. If there was any disagreement on this, we increased the degree of agreement through discussion.

**Figure 2:**
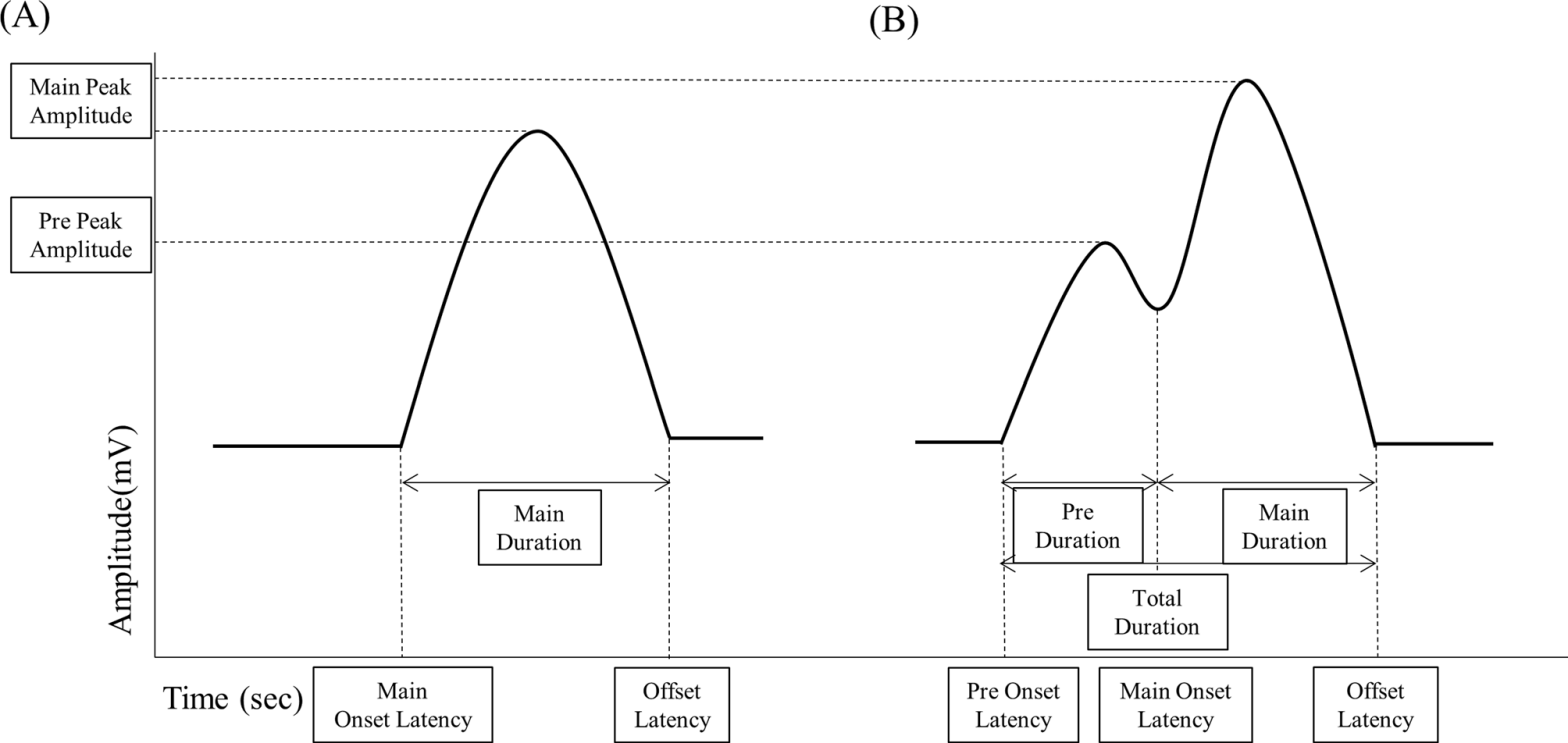
The two types of S-EMG activation patterns in the young and older groups. Type I pattern was characterized by a monophasic activation peak (**A**). Type II pattern was biphasic or triphasic, consisting of a pre-reflex peak and main peak (**B**).

The first type (type I) of muscle activation pattern was characterized by a monophasic shape consisting of only one peak during swallowing (Fig. 2A). The second type (type II) was a biphasic or triphasic pattern that consisted of several peaks. A common feature of muscle activation patterns with these multiple peaks is that the small peak develops and does not return to the resting state but continues to rise towards the maximum peak (Fig. 2B). These small peaks occur in swallowing-related muscles^15,20^, and were defined as pre-reflex peaks. Small peaks in the TH and StH muscles appeared only when peaks were already observed in the SH and RH muscles. Based on this, the type II patterns that had multiple peaks were further subdivided into three subgroups. Type II (a) was defined as a biphasic pattern only in the SH muscle, type II (b) was defined as the biphasic wave observed in the SH, RH, and TH muscles, and type II (c) was defined as the biphasic wave observed in all of the SH, RH, TH and StH muscles (Table 1).

**Table 1:** The classification the muscle activation patterns observed in oropharyngeal muscles during swallowing test.

	Monophasic	Biphasic
Type I	SH, RH, TH, StH	None
Type II(a)	TH, StH	SH, RH
Type II(b)	StH	SH, RH, TH
Type II(c)	None	SH, RH, TH, StH

SH, suprahyoid muscle; RH, retrohyoid muscle; TH, thyrohyoid muscle; StH, sternothyroid muscle.

To further analyze the patterns of the two types found in this study, the S-EMG signal was divided into the pre-reflex phase and the main phase, and each onset latency, duration, and peak amplitude values of each muscle were reanalyzed. To analyze the sequence of swallowing-related muscle contraction from swallowing initiation orders, the onset latency of the SH, which is the first activated muscle, was set to 0 as a reference point. Because the main phases of type I and type II were regarded as the same activation pattern, the pre-reflex phase of type II was defined as an activation that preceded the reference point and the onset latency was expressed as a negative value. Since there was no statistically significant difference between the SH and RH muscles, the mean values on both sides were used.

### Statistical analysis

Muscular activation sequences upon the swallowing of each liquid were compared between the older group and the younger group using the Mann Whitney U test. The Chi-square test was used to determine the cause of expression of the S-EMG activation patterns (type I or II), in relation to age and liquid characteristics. The Kruskal Wallis test was used to compare liquid characteristics within the same group. The Mann Whitney U test was used for post-hoc analysis. All statistical analyses were carried out using SPSS 22.0 software (SPSS Inc.; Chicago, IL, USA). A *p*-value of less than 0.05 was considered statistically significant.

## Results

### General observations

A total of 20 non-dysphagic older participants (> 60 years) and 20 young participants (< 60 years) were recruited. Their demographic data are presented in Table 2. There was no significant difference in the gender ratio between the two groups (*p* > 0.05).

**Table 2:** The classification and frequency of the muscle activation patterns observed in oropharyngeal muscles during swallowing test.

Age	Young group (< 60yrs)			Elderly group (> 60yrs)		
	43 ± 14 years			64 ± 4 years		
Gender	12 : 8			10 : 10		
	Water 2 cc	Water 5 cc	Yoplait 5 cc	Water 2 cc	Water 5 cc	Yoplait 5 cc
Type I	12	19	6	13	14	4
Type II(a)	3	1	1	1	2	0
Type II(b)	9	4	6	5	4	1
Type II(c)	10	12	25	17	15	30
Total	34	36	38	36	35	35

Since 20 participants in each group performed swallowing twice for each liquid, a total of 40 rounds were performed for each liquid. However, some baseline data were excluded due to the presence of considerable artifacts during the analysis. Therefore, the total number of analyzed trials for each liquid ranged between 34 and 40. However, the difference in the number of swallowing trials did not show a significant difference in both groups.

### Types of S-EMG activation patterns

**Figure 3:**
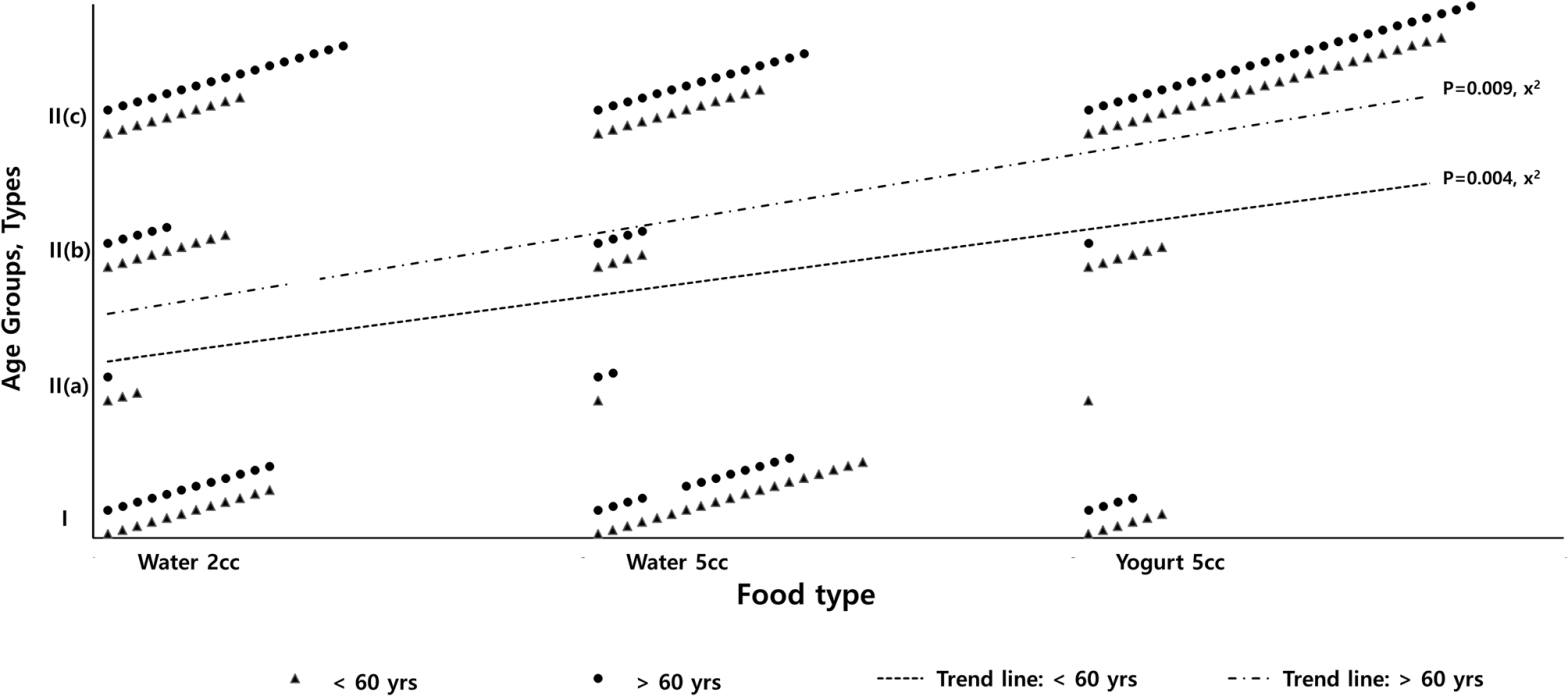
The distribution of S-EMG activation patterns according to the liquid characteristics in young and older groups. Trend lines in both groups show that the type II (c) pattern, which is defined as the biphasic wave, was observed in all of the SH, TH, and StH muscles and was more frequently observed when swallowing the 5 cc of yogurt in both groups.

There was no significant difference in the amplitude of the main peak between the two types. The frequency of the patterns observed when swallowing each liquid in the older and younger group are described in Table 2. The analysis between the S-EMG activation patterns (type I or II) and age showed no significant findings. However, the analysis between S-EMG activation pattern and liquid characteristics showed significant changes in the distribution pattern of liquid characteristics (*p* < 0.05). The type II pattern was more frequently observed in the swallowing of the highly viscous liquid (yogurt) compared to water, and when a larger volume (5 cc) of water was swallowed compared to a smaller volume (2 cc) (Fig. 3).

### Effects of fluid volume and viscosity on swallowing muscle activity

Quantitative results of muscle activation patterns according to volume and viscosity in type I are presented in Table 3. In type I, no significant difference was observed between swallowing 2 cc and 5 cc of water in the two groups. However, swallowing of a highly viscous liquid significantly prolonged the duration of activation of the SH, RH, TH, and StH muscles and increased the amplitude of the main peak of the RH muscle in the younger group. These changes were not observed in the older group (Table 3).

**Table 3:** Muscle activation duration and RMS values on type I pattern.

	Young group (< 60yrs)					Elderly group (> 60yrs)				
	Water 2 cc	Water 5 cc	Yoplait 5 cc	*p*^†^	*p*^‡^	Water 2 cc	Water 5 cc	Yoplait 5 cc	*p*^†^	*p*^‡^
**SH**										
Duration	1.439 ± 0.259	1.420 ± 0.247	1.934 ± 0.548	0.563	**0.001**	1.364 ± 0.186	1.473 ± 0.290	1.506 ± 0.054	0.243	0.070
Peak Amp	45.001 ± 27.237	41.310 ± 21.235	40.845 ± 9.262	0.828	0.212	46.375 ± 19.640	47.500 ± 19.682	61.079 ± 36.247	0.808	0.358
**RH**										
Duration	1.378 ± 0.172	1.447 ± 0.243	1.926 ± 0.588	0.193	**0.002**	1.407 ± 0.180	1.510 ± 0.343	1.586 ± 0.076	0.585	0.083
Peak Amp	22.784 ± 9.406	22.224 ± 7.083	30.978 ± 7.641	0.919	**0.001**	25.535 ± 14.454	25.189 ± 13.076	22.165 ± 2.769*	0.775	0.896
**TH**										
Duration	1.417 ± 0.362	1.347 ± 0.223	1.847 ± 0.457	0.735	**0.001**	1.255 ± 0.101	1.392 ± 0.328	1.477 ± 0.133	0.756	0.233
Peak Amp	28.537 ± 10.602	29.892 ± 13.293	33.517 ± 11.315	0.952	0.400	27.764 ± 8.640	31.628 ± 18.047	35.560 ± 16.579	0.943	0.505
**StH**										
Duration	1.356 ± 0.338	1.235 ± 0.326	1.763 ± 0.493	0.269	**0.014**	1.108 ± 0.197*	1.292 ± 0.334	1.461 ± 0.166	0.169	0.158
Peak Amp	15.668 ± 5.116	14.827 ± 6.413	15.230 ± 6.939	0.484	0.733	15.526 ± 5.044	16.78 ± 8.776	21.413 ± 5.496	0.943	0.233

SH; suprahyoid, RH; retrohyoid, TH; thyrohyoid, StH; sternothyroid, Amp; Amplitude.

*p*^†^; *p*-value between water 2 cc and 5 cc swallowing in same group, *p*^‡^; *p*-value between water 5 cc and yogurt 5 cc swallowing in same group.

*: The *p*-value < 0.05 between young group and elderly group in each food types.

There were significant differences in type II between the young and older groups. Type II results are presented in Table 4. In the younger group, the type II findings were concordant with those of type I. There was no significant difference in swallowing between 2 and 5 cc of water; however, the highly viscous fluid significantly prolonged the activation duration of the SH, RH, TH, and StH muscles, and significantly increased the amplitude of the pre-reflex peak and main peak of the SH and RH muscles, and the pre-reflex peak of the TH and StH muscles in the younger group (Table 4). A type II pattern during the swallowing of the highly viscous fluid among the older group also significantly prolonged the activation duration of the SH, RH, TH, and StH muscles and significantly increased the amplitude of the pre-reflex peak of the SH, RH, and TH muscles (Table 4).

**Table 4:** Difference of S-EMG variables of type II pattern depending on liquid characteristics in same group.

	Young group (< 60yrs)					Elderly group (> 60yrs)				
	Water 2 cc	Water 5 cc	Yoplait 5 cc	*p*^†^	*p*^‡^	Water 2 cc	Water 5 cc	Yoplait 5 cc	*p*^†^	*p*^‡^
**SH**										
Pre_Dur	0.456 ± 0.183	0.412 ± 0.098	0.645 ± 0.290	0.506	**0.000**	0.590 ± 0.326*	0.646 ± 0.313*	0.735 ± 0.406	0.267	0.131
M_Dur	1.206 ± 0.205	1.178 ± 0.224	1.341 ± 0.247	0.506	**0.001**	1.028 ± 0.280*	1.135 ± 0.3334	1.343 ± 0.517	0.064	**0.001**
Tot_Dur	1.662 ± 0.241	1.590 ± 0.194	1.987 ± 0.328	0.249	**0.000**	1.617 ± 0.411	1.781 ± 0.511	2.078 ± 0.787	0.116	**0.001**
Pre_Pk_Amp	13.248 ± 5.754	11.557 ± 3.800	20.874 ± 16.359	0.292	**0.000**	23.618 ± 20.779*	26.645 ± 28.491*	36.375 ± 27.543*	0.581	**0.007**
M_Pk_Amp	34.466 ± 14.539	31.689 ± 11.197	42.933 ± 19.924	0.569	**0.002**	47.772 ± 33.483	46.546 ± 35.100*	54.230 ± 30.048*	0.993	0.062
**RH**										
Pre_Dur	0.352 ± 0.373	0.408 ± 0.093	0.584 ± 0.261	0.583	**0.001**	0.520 ± 0.242*	0.581 ± 0.273*	0.68 ± 0.398*	0.268	0.282
M_Dur	1.168 ± 0.423	1.157 ± 0.214	1.305 ± 0.241	0.672	**0.003**	1.070 ± 0.288*	1.176 ± 0.369	1.332 ± 0.519	0.156	**0.003**
Tot_Dur	1.520 ± 0.400	1.565 ± 0.201	1.944 ± 0.361	0.632	**0.000**	1.590 ± 0.332	1.757 ± 0.502	2.083 ± 0.783	0.116	**0.000**
Pre_Pk_Amp	9.678 ± 4.634	7.328 ± 2.983	11.455 ± 5.828	**0.024**	**0.000**	11.171 ± 8.829	11.953 ± 8.030*	20.139 ± 15.593	0.380	**0.005**
M_Pk_Amp	21.722 ± 12.686	18.600 ± 8.832	24.346 ± 14.987	0.323	**0.035**	21.599 ± 11.286	19.516 ± 9.790	28.061 ± 14.218	0.425	**0.002**
**TH**										
Pre_Dur	0.400 ± 0.172	0.428 ± 0.078	0.451 ± 0.244	0.125	0.840	0.562 ± 0.293*	0.541 ± 0.224	0.521 ± 0.336	0.748	0.697
M_Dur	1.186 ± 0.186	1.156 ± 0.204	1.320 ± 0.228	0.756	**0.016**	0.945 ± 0.211*	1.188 ± 0.360	1.343 ± 0.514	**0.015**	**0.039**
Tot_Dur	1.586 ± 0.236	1.584 ± 0.203	1.488 ± 0.316	0.857	0.318	1.507 ± 0.379	1.728 ± 0.484	1.537 ± 0.617	0.215	0.497
Pre_Pk_Amp	10.168 ± 4.296	8.414 ± 2.338	12.282 ± 6.214	0.317	**0.015**	12.173 ± 10.409	11.080 ± 4.176*	19.487 ± 13.928*	0.469	**0.017**
M_Pk_Amp	30.652 ± 12.668	28.993 ± 10.380	28.899 ± 10.629	0.961	1.000	25.988 ± 7.573	28.970 ± 8.679	33.440 ± 11.853	0.347	0.219
**StH**										
Pre_Dur	0.349 ± 0.187	0.382 ± 0.081	0.397 ± 0.296	0.456	0.109	0.535 ± 0.214*	0.428 ± 0.305	0.429 ± 0.358	0.123	0.838
M_Dur	1.244 ± 0.164	1.067 ± 0.237	1.314 ± 0.245	0.093	**0.013**	0.876 ± 0.146*	1.160 ± 0.384	1.281 ± 0.507	**0.009**	0.123
Tot_Dur	1.592 ± 0.253	1.448 ± 0.236	1.837 ± 0.355	0.159	**0.001**	1.412 ± 0.279	1.587 ± 0.511	1.969 ± 0.711	0.433	**0.007**
Pre_Pk_Amp	8.869 ± 6.197	5.779 ± 1.447	9.278 ± 4.921	0.346	**0.008**	7.587 ± 3.939	6.965 ± 3.229	11.155 ± 7.875	0.882	0.087
M_Pk_Amp	16.005 ± 5.218	15.037 ± 4.861	16.778 ± 6.249	0.771	0.491	14.262 ± 6.450	14.589 ± 6.182	16.356 ± 8.049	0.794	0.613

SH; suprahyoid, RH; retrohyoid, TH; thyrohyoid, StH; sternothyroid, Amp; Amplitude, Pre_Dur; pre-reflex duration, M_Dur; main reflex duration, Tot_Dur; total duration, Pre_Pk_RMS; pre-reflex peak amplitude by root mean square, M_Pk_RMS; main-reflex peak amplitude by root mean square,

*p*^†^; *p*-value between water 2 cc and 5 cc swallowing in same group, *p*^‡^; *p*-value between water 5 cc and yogurt 5 cc swallowing in same group.

*: The *p*-value < 0.05 between young group and elderly group in each food types.

In the younger group, the amplitude of the pre-reflex peak of the SH, TH, and StH muscles decreased as the amount of fluid increased from 2 to 5 cc, with a significant difference observed in the RH muscles (*p* < 0.05). In the older group, however, the amplitude of the pre-reflex peak of the SH and RH muscles increased as the amount increased from 2 to 5 cc (Table 4).

### Effect of age on swallowing muscle activity

**Figure 4:**
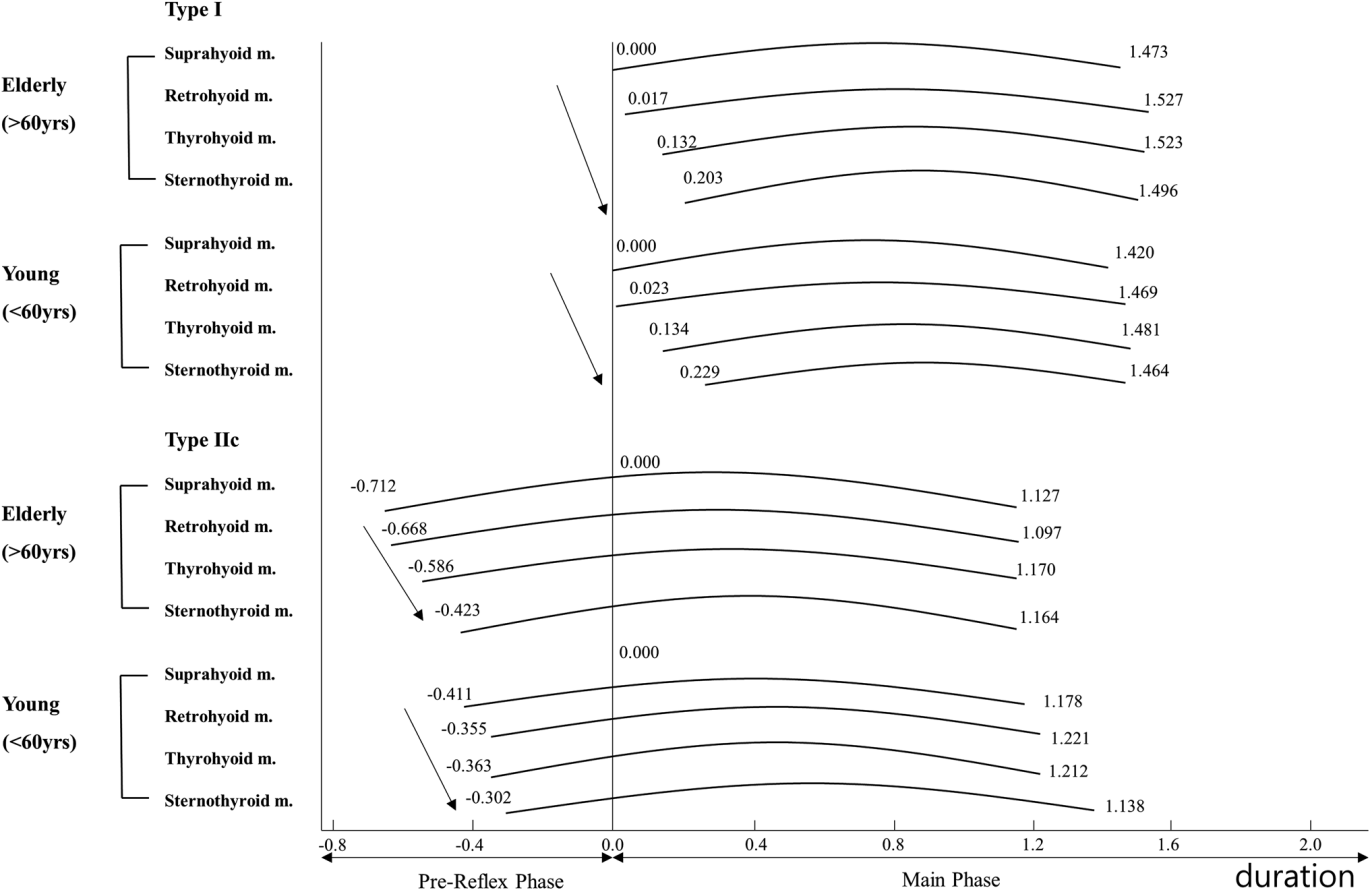
The activation sequence of oropharyngeal muscles during 5 cc water swallowing. S-EMG revealed that the activation sequence was in the order of the suprahyoid (SH), retrohyoid (RH), thyrohyoid (TH), and sternothyroid (StH) muscles in both the young and older groups.

From the perspective of sequential activation of the pharyngeal muscles, activation sequences were generally in the order of the SH, RH, TH, and StH muscles (Fig. 4).

Comparison of age-related changes in each type of pattern was performed. In type I, only the amplitude of the main peak of the RH muscle during the swallowing of highly viscous fluids and the activation duration of the StH muscle during the swallowing of 2 cc of water were significantly different between the two groups. All other parameters related to the swallowing of all liquid types were not significantly different between the two groups (Table 3, *p < 0.05, bold characters).

On the other hand, statistically significant differences were observed with a constant pattern in type II. In the 2 cc water swallowing task, the older group showed a significant increase in the duration of the pre-reflex phase of the SH, RH, TH, and StH muscles and the amplitude of the pre-reflex peak of the SH muscle, compared to those in the younger group. Swallowing of 5 cc of water showed similar results regarding the duration of the pre-reflex phase of the SH and RH muscles and the amplitude of the pre-reflex peak of the SH, RH, and TH muscles. Swallowing of 5 cc of the highly viscous fluid showed similar results regarding the duration of the pre-reflex phase of the RH muscle and the amplitude of the pre-reflex peak of the SH and TH muscles (Table 4, *p < 0.05, bold characters).

## Discussion

The purpose of this study was to understand changes in the activation pattern of the oropharyngeal muscle that are associated with aging. In this study, we observed changes in the activation patterns of the oropharyngeal muscles according to the aging process. The findings indicate that the oropharyngeal muscles are activated from the SH muscles to the infrahyoid muscles, regardless of age and the type of liquid being swallowed (Fig. 4). The oropharyngeal muscle, particularly the SH muscles, was found to develop a pre-reflex phase with increasing volume and viscosity of the swallowed fluid. In addition, a significant increase in the duration and amplitude of the pre-reflex phase was observed in the older group compared to those in the younger group.

The physiological swallowing reaction is controlled by the modulation of the swallowing motor response according to sensory input from the oropharyngeal sensory receptors^21,22^. Previous studies have revealed a sequence of oropharyngeal motor responses in which the infrahyoid muscle is activated after initial SH muscle activation. Matched with this activation sequence, the hyoid bone begins with an upward movement, performs circular rhythmic movements, and correlates with the UES opening time^19,23^. The results of this study also confirmed that oropharyngeal muscles are activated from the SH muscles to the infrahyoid muscles, regardless of the aging process.

In addition, the present study selectively distinguished the pre-reflex phase of the oropharyngeal muscles via the shape of the S-EMG. We also reported the frequency and propensity of the pre-reflex activation pattern (Table 1). All 214 swallowing tasks from the 40 participants appeared to have a consistent muscle activation pattern, with no exceptions (Table 2). Because the duration and amplitude of the main phases of type I and type II patterns are similar, the small-sized peaks that appear before the main phase of type II are considered to constitute the pre-reflex phase.

In this study, the duration of the main phase in type II was slightly shorter compared to that in type I; the reason could be that we could not identify the initiation point of the main phase in type II. We estimated that the time of oropharyngeal muscle contraction and hyoid bone movement occurred simultaneously, which was referred to as the "main-reflex" phase. Furthermore, the preparatory stage just before the “main-reflex” phase was referred to as the “pre-reflex” phase. In particular, referring to a previous study, the onset latency of the SH and infrahyoid muscles occurs faster than hyoid bone motion and continues until hyoid bone motion is terminated^19,23^. Several previous studies have been reported to support this. Zhu et al. reported that multiple peaks occur upon intake of highly viscous fluids in a high-density S-EMG study; however, they estimated this to be due to multiple retries^20^. However, a review by Vaiman et al. reported that despite multiple peaks of muscle activation and increased muscle effort, the duration of swallowing responses did not change^15^. Based on these studies and our findings, we can hypothesize that the small peaks reflect effortful muscle activation prior to the main-reflex phase of swallowing. We further hypothesized that this activation may be related with the isometric contraction of oropharyngeal muscles. However, this hypothesis was not specifically addressed in this study. The exact mechanism of the pre-reflex phase is uncertain; however, it is suspected to be related to the protective mechanism of swallowing^24^. To the best of our knowledge, this is the first report on the pre-reflex phase of the oropharyngeal muscles and their respective patterns. Classification of the muscle activation patterns, including the pre-reflex phase, can be used to investigate the physiology, contractile interval, and protective mechanisms of swallowing in future studies.

Aging is accompanied with anatomical changes, such as atrophy of the oropharyngeal muscles, including the geniohyoid muscles, decreased elasticity of the hypoepiglottic ligaments, and decreased UES compliance^25,26^. Due to these anatomical changes, the range of motion and velocity of the hyoid bone are kinematically reduced, which is closely related to the decrease in the UES opening time^11,27^. A reduction in the UES opening time results in increased penetration, aspiration, and pharyngeal residue of the food material during a bolus transit^28^. However, little is known about the neurological and adaptational changes related to the aging process. In previous studies using kinematic analysis, besides the delay in the swallow reaction time (bolus entering the pharynx to onset of hyoid excursion) and the decrease in hyoid bone movement velocity, other parameters such as transit time are preserved in normal older people^11,16^. However, these studies used a VFSS, which evaluates the motion of the hyolaryngeal structure in a functional manner, and therefore has a limitation in directly evaluating individual oropharyngeal muscles. In addition, needle or S-EMG analyses were performed in previous studies in limited oropharyngeal muscles related to swallowing^29–32^.

In the present study, the older group responded differently to bolus volume and viscosity changes in type I and II patterns. For example, the swallowing of more a viscous liquid significantly prolonged the activation duration of the SH (*p* = 0.001), RH (*p* = 0.002), TH (*p*  = 0.001), and StH muscles (*p*  = 0.014) and increased the peak amplitude of the RH muscle (*p*  = 0.001) in the type I pattern in the younger group, but not in the older group (*p*  > 0.05). Furthermore, the amplitude of the pre-reflex peak value of all the SH, RH, TH and StH muscles decreased as the amount of fluid increased from 2 to 5 cc in the type II pattern in the younger group. However, statistical significance was found only in the RH muscle (*p*  = 0.024). On the contrary, the amplitude of the pre-reflex peak values of the SH and RH muscles increased as the amount of fluid increased from 2 to 5 cc in the older group (*p*  > 0.05). The possible reason for the observed significance in the younger group might be the sufficient generation of pressure and a functionally normal control system. It has been established that swallowing process in a healthy population can be adapted by varying the pressure and timing to change the viscosity of the liquid., which was proven by several studies with kinematic and pressure analyses^33,34^. The s-EMG pattern in the older group could be associated with difficulty in generating forces or reduced oral sensory perception^22,33^. It is known that a four-fold increase in liquid volume is required by older participants to perceive an approximate two-fold increase in the perception of volume compared with that in younger healthy adults^22^. The further research is needed in this aspect.

From a different perspective, the present study showed the different responses between type I and II patterns in the older group. Though the type I pattern in the older group did not show any changes in response to changes in the bolus volume and viscosity, the type II pattern in the older group had a significantly prolonged the main duration of the SH, RH, TH, and StH muscles, and a significantly increased amplitude of the pre-reflex peak of the SH, RH, and TH muscles. These responses in the type II pattern among the older group are concordant with those observed in the younger group. What is certain is that in the type II pattern, the older participants reacted in a manner that was more similar to the younger group than in the type I pattern. In other words, the pre-reflex phase assists the swallowing process of the older group in order for it to function like that of the younger group, or the pre-reflex phase may be an adaptation process to compensate for the aging process to prevent aspiration.

This study has a limitation in relation to the crosstalk phenomenon. The summation of activation signals of muscles around the electrode attachment site cannot be excluded due to the characteristics of S-EMG. However, we considered that no significant error occurred because the researchers cited and used the ultrasound-guided electrode positioning method used in a previous study^19^. When we planned this study, we were concerned about the accuracy of S-EMG in evaluating the oropharyngeal muscles, which are small and closely positioned, increasing the crosstalk phenomena. However, a previous study showed that the graphic record for S-EMG is a valid and reliable tool for identifying normal physiological swallowing and has a good inter-judge agreement^35^. Our results regarding the order of the activation sequence being the SH, RH, TH, and StH muscles were concordant with those of a previous study, which used needle EMG^19^. Therefore, an S-EMG study performed with an anatomical basis maybe as reliable as a needle EMG study when detecting the latency, amplitude, and activation duration of the oropharyngeal muscles. Second, we performed only S-EMG analysis without VFSS ; thus, this study is solely focused on muscle activation without taking into account the location of the bolus or the movement of structures in the oral cavity or pharynx in relation to one another. Third limitation is that the study used only a small amount of liquid. Therefore, a further study with larger volumes will be needed in the future.

## Conclusion

In the present study, the S-EMG activation patterns of the oropharyngeal muscles were classified as type I and type II (a), (b), and (c) according to the pre-reflex phase and its occurrence pattern. The activation sequences measured with S-EMG were in the order of the SH, RH, TH, and StH muscles, which were concordant with previously reported needle EMG results. The oropharyngeal muscles, particularly the SH muscles, were found to develop a pre-reflex phase specifically with increasing volume and viscosity of the swallowed fluid. In addition, a significant increase in the duration and amplitude of the pre-reflex phase was observed in the older group compared to that in the younger group.

The present study showed the changes in the activation patterns of oropharyngeal muscles in accordance with the aging process. Specifically, the type I pattern in the older group showed a different response to highly viscous fluid compared to that in the younger group; however, the type II pattern in the older group responded concordantly to that in the younger group. Therefore, healthy older people were found to compensate swallowing with a pre-reflex phase of muscle activation in response to increased liquid volume and viscosity to adjust for age-related muscle weakness.
